# Serum Levels of Cytokine-Induced Apoptosis Inhibitor 1 (CIAPIN1) as a Potential Prognostic Biomarker of Cholangiocarcinoma

**DOI:** 10.3390/diagnostics11061054

**Published:** 2021-06-08

**Authors:** Son Dinh An Truong, Doungdean Tummanatsakun, Tanakorn Proungvitaya, Temduang Limpaiboon, Molin Wongwattanakul, Daraporn Chua-on, Sittiruk Roytrakul, Siriporn Proungvitaya

**Affiliations:** 1 Centre of Research and Development of Medical Diagnostic Laboratories, Faculty of Associated Medical Sciences, Khon Kaen University, Khon Kaen 40002, Thailand; sontda@kkumail.com (S.D.A.T.); pui_ddlab41@hotmail.com (D.T.); tanakorn@kku.ac.th (T.P.); temduang@kku.ac.th (T.L.); moliwo@kku.ac.th (M.W.); chuaondaraporn@kkumail.com (D.C.-o.); 2 Cholangiocarcinoma Research Institute, Faculty of Medicine, Khon Kaen University, Khon Kaen 40002, Thailand; 3 Center for Innovation and Standard for Medical Technology and Physical Therapy, Faculty of Associated Medical Sciences, Khon Kaen University, Khon Kaen 40002, Thailand; 4 National Center for Genetic Engineering and Biotechnology, National Science and Technology Development Agency, Pathumthani 12120, Thailand; sittiruk@biotec.or.th

**Keywords:** CIAPIN1, serum, dot blot, prognostic biomarker, cholangiocarcinoma

## Abstract

The mortality rate of cholangiocarcinoma (CCA) is high since there is a lack of a non-invasive technique to accurately detect tumors at the early stage. CCA biomarkers are consistently needed for various purposes including screening, early diagnosis, prognosis and follow-up. Herein, using bioinformatic analysis of our mitochondrial proteome database of CCA tissues, we identified cytokine-induced apoptosis inhibitor 1 (CIAPIN1) as a potential prognostic biomarker for CCA. CIAPIN1 levels in the sera of 159 CCA patients and 93 healthy controls (HC) were measured using a dot blot assay. The median level ± quartile deviation of CIAPIN1 level in the sera of CCA patient group was 0.5144 ± 0.34 µg/µL, which was significantly higher than 0.2427 ± 0.09 µg/µL of the HC group (*p* < 0.0001). In CCA patients, higher serum CIAPIN1 level was significantly associated with lymph node metastasis (*p* = 0.024) and shorter overall survival time (*p* = 0.001, Kaplan–Meier test). Cox regression analysis showed that the serum CIAPIN1 level can be an independent prognostic indicator for the survival of CCA patients. Moreover, for the prediction of CCA prognosis, CIAPIN1 is superior to CEA, CA19-9 and ALP. In conclusion, CIAPIN1 can be a serum biomarker candidate for the poor prognosis of CCA.

## 1. Introduction

Cholangiocarcinoma (CCA) is a highly aggressive malignant tumor originating from any point of the biliary tree. CCA accounts for approximately 15% of all primary liver cancers and is the cause of 2% of cancer-related death [1]. The incidence of CCA is especially high in northeastern Thailand in association to the high incidence of infestation with the carcinogenic liver fluke, *Opisthorchis viverrini* (OV), which is the major risk factor for CCA [2]. CCA is usually asymptomatic in the early stage and, therefore, often diagnosed only when the disease has already been in the advanced stage, which highly compromises therapeutic options, resulting in a dismal prognosis [3]. Diagnosis of CCA is usually conducted by a combination of clinical, biochemical, radiological, and histopathological information. Reliable diagnostic/prognostic markers are critically necessary for CCA treatment. Currently, several CCA markers, such as carbohydrate antigen 19–9 (CA19-9), carcinoembryonic antigen (CEA) and alkaline phosphatase (ALP), are the most widely clinically used biomarkers that have been applied for diagnosis, prognostic stratification, and treatment prediction. Nonetheless, their specificity and sensitivity are variable and not quite satisfactory, even when the combination of those markers is used for CCA diagnosis and prognosis [4,5,6,7,8]. Hence, good biomarkers are urgently needed to identify CCA patients with poor prognosis who may benefit from more aggressive surgical strategies and effective management.

Various biomolecules, such as nucleic acids, proteins and glycoproteins, including several mitochondrial proteins associated with CCA progression, have been discovered and suggested as candidates for prognostic markers in CCA. The vast majority of mitochondrial proteins are encoded by nuclear genes, synthesized in cytosol, and moved into proper sub-compartments of mitochondria [9]. Thus, mitochondrial proteins can be detected not only in mitochondria but also in the cytoplasm and nucleus. In our previous study, using mass spectrometry, Chua-On et al. identified a total of 281 mitochondrial proteins in cancerous and adjacent non-cancerous tissues of CCA [10]. Among them, several mitochondrial proteins, such as apoptosis-inducing factor, mitochondrion-associated 3 (AIFM3) and pyruvate dehydrogenase kinase 3 (PDK3), have been detected in the serum, and are suggested to be potential prognostic markers for CCA [11,12]. In this study, therefore, we re-analyzed our mitochondrial proteins of CCA tissues by using a series of bioinformatics analysis, such as diagram interaction, differential expression, survival correlation and secretory protein nature, to screen and identify the selected candidate. The candidate was validated, and measured its levels in the sera of healthy controls and CCA patients. Then, the correlation between its expression and clinical features was conducted to determine novel prognosis biomarkers for CCA. Here, we report that CIAPIN1 was identified as a serum biomarker candidate for the poor prognosis of CCA.

## 2. Materials and Methods

### 2.1. Sample Size Calculation

In the preliminary study using a dot blot assay, the mean and standard deviation values of CIAPIN1 levels of 23 CCA and 23 healthy control (HC) sera were determined. Then, these values of the two groups were used for sample size calculation. Using G*Power program version 3.1.9.4, the G*Power team, Heinrich Heine Universität, Düsseldorf, Germany [13], the minimum sample size necessary for comparison of the mean between the CCA group and HC group was determined to be 88 samples.

### 2.2. Sera from CCA Patients and HC

Preoperative serum samples from 159 CCA patients who were diagnosed as having intrahepatic CCA (median age ± quartile deviation, 61 ± 6.5 years; range 31 to 78 years) were provided by the Cholangiocarcinoma Research Institute (CARI), Faculty of Medicine, Khon Kaen University, Thailand. The inclusion criteria were the patients being diagnosed as having intrahepatic CCA by clinicopathological examinations. The patients were subjected to preoperative and postoperative care without receiving any chemotherapeutic treatment. The exclusion criteria were those who were diagnosed as having extrahepatic CCA or hepatocellular carcinoma. The HC samples were leftover sera from 93 healthy persons (median age ± quartile deviation, 41 ± 8 years; range 19 to 85 years) who received the annual health check-up in the Faculty of Associated Medical Sciences (AMS-KKU Excellence Laboratory), Khon Kaen University, Thailand, with a generally healthy appearance and normal biochemical tests for liver and kidney functions. The samples were kept at −80 °C until use. All subjects gave their informed consent to the CARI for reuse of their specimen for the research purpose. The study was conducted in accordance with the Declaration of Helsinki, and the protocol was approved by the Ethics Committee of Khon Kaen University, Thailand (HE631387).

### 2.3. Bioinformatic Analysis in Identification of Potential Prognosis Biomarker

Firstly, to compare mitochondrial protein expressions in CCA and the adjacent normal tissue of our database, we used jvenn, which is an open-source component in the web environment [14]. Secondly, three bioinformatics software programs were used to predict the secretory protein nature of the candidate molecules, including: (i) SignalP software ver.4.1 servers (Department of Bio and Health informatics, Technical University of Denmark, Lyngby, Denmark, accessed on 19 December 2020) to predict the signal peptide cleavage site in the amino acid sequence using D-score > 0.45 for the presence of a signal peptide within a protein sequence [15], (ii) SecretomeP software ver. 2.0 server (Department of Bio and Health Informatics, Technical University of Denmark, Lyngby, Denmark, accessed on 19 December 2020) to predict proteins with a neural network score (NN score) > 0.5 for confirmation of secretory proteins via an unconventional secretion pathway without a signal peptide [16], and (iii) Plasma Proteome Database (PPD) Version 2014 (under the Human Proteome Organizations, accessed on 19 December 2020) to identify proteins present in serum or plasma [17]. Then, to see the mRNA expression levels of candidate proteins in CCA and normal, and their correlation with survival time of the CCA patients, we used the open-access internet database of Gene Expression Profiling Interactive Analysis (GEPIA 2, http://gepia2.cancer-pku.cn/, Peking University, Beijing, China, accessed on 19 April 2021) [18]. A *p*-value < 0.05 was used to compare the expression levels between CCA and HC groups, whereas *p* < 0.1 was used to examine the correlation between the expression levels and the overall survival time for the statistical significance.

### 2.4. Western Blot Analysis

Fifty micrograms of protein of each of three randomly selected HC and CCA sera were dissolved in sample buffer (10% sodium dodecyl sulfate (SDS), 1 M Tris-HCl, pH 6.8), and boiled for 5 min. The samples were separated on 12.5% SDS-PAGE at 150 V for 2 h at room temperature. The samples were loaded and run in parallel with standard molecular weight markers. After electrophoresis, proteins were transferred electrically onto the PVDF membrane (GE Healthcare Life Sciences, Little Chalfont, UK) for 1 h at room temperature. The membrane was blocked with 5% skim milk in Tris-buffered saline with 0.1% Tween-20 (1× TBST, pH 7.4) for 1 h at room temperature. The membrane was then incubated with 1:1000 dilution of rabbit polyclonal antibody against human CIAPIN1 (Cat#orb377996, Biorbyt, Cambridge, UK) overnight at 4 °C. The membrane was washed with 1× TBST, incubated with 1:10,000 dilution of horseradish peroxidase-conjugated goat anti-rabbit IgG secondary antibody for 1 h at room temperature, and washed with 1× TBST. Finally, peroxidase activity was detected as chemiluminescence using enhanced chemiluminescence (ECL) plus reagent (GE Healthcare Life Sciences, Buckinghamshire, UK) and quantitatively analyzed using an Amersham imager 600 (GE Healthcare Bio-Sciences AB, Uppsala, Sweden).

### 2.5. Dot Blot Assay and Acquisition of Data

The nitrocellulose membrane (GE Healthcare Life Sciences, Little Chalfont, UK) was soaked in 1× TBST for 10 min before placing on the spotting machine. HC sera were undiluted. Each CCA serum sample was diluted at 1:2 with normal saline (NSS). Two µL each was spotted onto the membrane using a Bio-Dot microfiltration apparatus (Bio-Rad Laboratories, Inc., Hercules, CA, USA). For each assay, the pooled CCA sera were used as a positive control. Then, the membrane was soaked in 5% skim-milk in 1× TBST for 1 h at room temperature to block nonspecific binding. The membrane was then incubated with 1:1000 dilution of the primary antibody (rabbit polyclonal antibody against human CIAPIN1) (Cat#orb377996, Biorbyt, Cambridge, UK) overnight at 4 °C. The membrane was washed with 1× TBST and then incubated with 1:10,000 dilution of horseradish peroxidase-conjugated goat anti-rabbit IgG secondary antibody for 1 h at room temperature and washed with 1× TBST. The chemiluminescent signal was detected using ECL plus reagent (GE) and quantified on an Amersham imager 600. The dot blot result (each spot) was computed for CIAPIN1 intensity by ImageJ software v.1.52d (National Institute of Health, Bethesda, MD, USA). The experiment was performed in triplicate. To prepare a standard curve, recombinant CIAPIN1 protein (Cat#orb244859, Biorbyt, Cambridge, UK) with a known concentration (2.44 µg/µL) was diluted twofold as, 1.22, 0.61, 0.305, and 0.1525 µg/µL, respectively. The intensities of CIAPIN1 protein in the sera were normalized using CIAPIN1 intensity in a positive control as a relative expression [19]. Subsequently, the relative expression of CIAPIN1 in each serum sample was calculated based on the standard curve prepared using the standard recombinant CIAPIN1 protein.

### 2.6. Statistical Analysis

The mean ± standard deviation or the median ± quartile deviation with the range (minimum to maximum) were used for the description of normally and non-normally distributed data, respectively [20]. The different values between two independent sample groups were estimated using the Mann–Whitney U test. The associations between serum CIAPIN1 levels and patients’ clinicopathological parameters were analyzed using Fisher’s exact test. The correlation between two variables was analyzed by the Spearman’s correlation test. The overall survival curves were analyzed using the Kaplan–Meier method and log-rank test. The Cox proportional hazards regression model was used for univariate and multivariate analyses. *p* < 0.05 was considered statistically significant. IBM SPSS ver.26 software (IBM Corp., Armonk, NY, USA) was used for statistical analyses.

## 3. Results

### 3.1. Bioinformatic Analysis of Mitochondrial Protein Database and CIAPIN1 Selection

**Figure 1:**
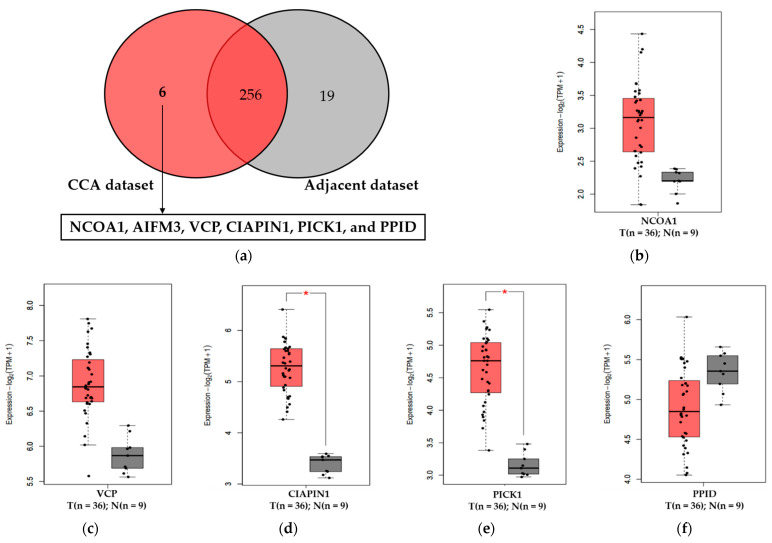
Expression of five selected mitochondrial proteins in CCA and normal tissues. (**a**) Venn-diagram demonstrating six mitochondrial proteins uniquely expressed in CCA dataset. (**b–f**) Gene expression levels of five candidate proteins in CCA and normal tissues were retrieved from the GEPIA2 database. NCOA1 (nuclear receptor coactivator 1 isoform 3), AIFM3 (apoptosis-inducing factor, mitochondrion-associated 3), VCP (transitional endoplasmic reticulum ATPase), CIAPIN1 (cytokine-induced apoptosis inhibitor 1), PICK1 (PRKCA-binding protein), and PPID (peptidyl-prolyl cis-trans isomerase D), T: tumor (red box), N: normal (grey box). * Statistically significant difference with *p* < 0.01.

**Figure 2:**
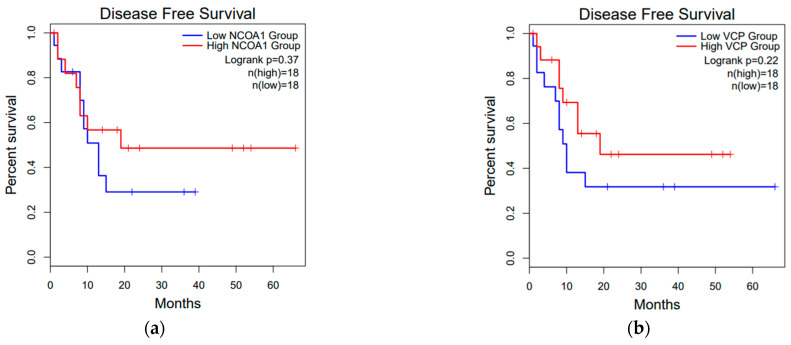

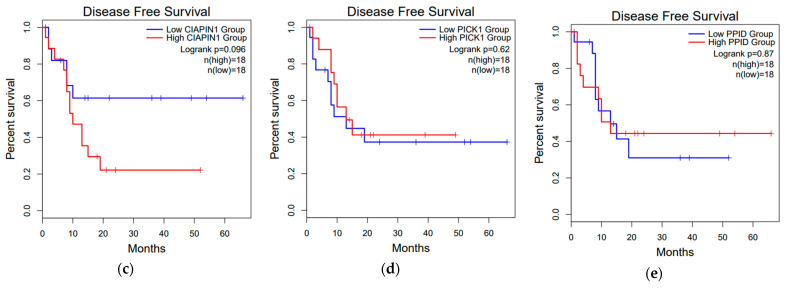
The prognostic information of the five selected mitochondrial proteins. The GEPIA2 online open-access gene expression database was used to see the prognostic value of mRNA expression levels of (**a**) NCOA1, (**b**) VCP, (**c**) CIAPIN1, (**d**) PICK1, and (**e**) PPID, and, out of five candidates, only CIAPIN1 was correlated significantly (*p* < 0.1) with worse disease-free survival.

To focus on mitochondrial proteins that were expressed uniquely in CCA tissues, 281 mitochondrial proteins [10] were divided into cancerous and adjacent non-cancerous groups and were analyzed using the jvenn’s web application (Figure 1a). The Venn diagram indicated six CCA-related mitochondrial proteins, including nuclear receptor coactivator 1 isoform 3 (NCOA1), apoptosis-inducing factor, mitochondrion-associated 3 (AIFM3), transitional endoplasmic reticulum ATPase (VCP), CIAPIN1, PRKCA-binding protein (PICK1), and peptidyl-prolyl cis-trans isomerase D (PPID). Among those, AIFM3 was excluded due to it already being detected in the sera of CCA and HC [12]. Tools in the Gene Expression Profiling Interactive Analysis 2 (GEPIA2) databases were used to evaluate the expression levels of the genes of these five proteins unique in CCA (Figure 1b–f). The results showed that CIAPIN1 and PICK1 in the CCA cancer type were overexpressed compared with the normal tissue (Figure 1d,e, *p* < 0.01). Then, we investigated the relationship between gene expression levels of these five candidate proteins and disease-free survival of CCA patients. As shown in Figure 2, of five candidates, only CIAPIN1 showed a significant association of higher mRNA expression with the shorter disease-free survival of CCA patients (Figure 2c, *p* < 0.1). These results suggested that CIAPIN1 might be a potential biomarker to predict the prognosis of CCA patients.

**Figure 3:**
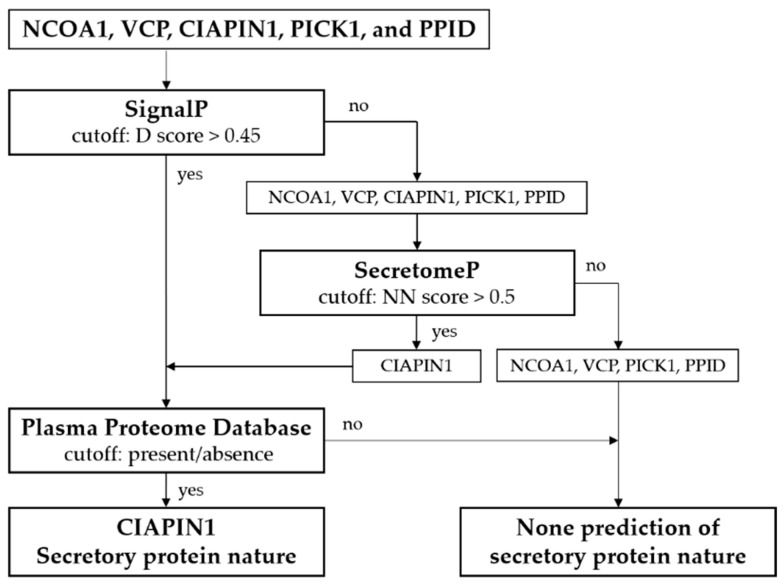
Bioinformatic analysis of secretory proteins identified uniquely CIAPIN1 as a secreted protein.

Since the peripheral venous blood is a minimally invasive sample, a promising biomarker should be present in it. Hence, bioinformatic analyses of secretory protein nature were performed to predict whether five selected candidates can be present in plasma/serum (Figure 3). Firstly, using SignalP software 4.1, the result showed that none of them have signal peptides or cleavage sites. Then, SecretomeP 2.0 was used to analyze possible secretion via a non-classical pathway. The results showed that only the NN score of CIAPIN1 (0.639) was significant. Finally, the presence of candidate proteins in plasma/serum was consulted to PPD. The results illustrated that CIAPIN1 was listed in PPD. Taken together, the results indicated that CIAPIN1 could be a potential predictive biomarker in the sera of CCA patients. By now, no article has been reported on the level of CIAPIN1 in the sera of healthy or diseased subjects including CCA; hence, CIAPIN1 was selected for the present study.

### 3.2. Serum CIAPIN1 Levels of CCA and HC

**Figure 4:**
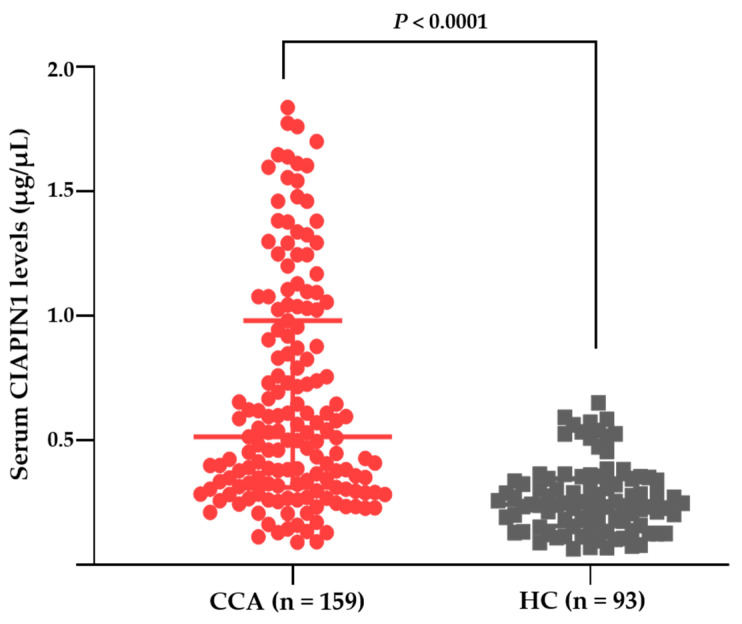
The serum CIAPIN1 levels of CCA and HC groups using a dot blot assay. The median level ± quartile deviation of CIAPIN1 was 0.5144 ± 0.34 µg/µL in the CCA group and 0.2427 ± 0.09 µg/µL in the HC group.

The characteristics of 159 CCA patients and 93 healthy controls in this study were shown in Table 1. To check the applicability and specificity of anti-CIAPIN1 antibody and validate whether CIAPIN1 protein can be detected in the sera of CCA patients, three sera each were selected randomly from HC and CCA groups, and its expression levels were examined using Western blot analysis. The results illustrated the clear appearance of approximately 38 kDa size band, which is the expected molecular size of CIAPIN1 (Figure S1). Then, we used this antibody for the quantitative measurement of CIAPIN1 protein levels of 159 CCA and 93 control sera using a dot blot assay and its standard curve. The standard curve of CIAPIN1 was created using a commercially available recombinant CIAPIN1 protein (Figure S2). As shown in Figure 4, the median CIAPIN1 level of the sera of CCA patients was significantly higher than that of HC. A representative dot blot image is presented in the Supplementary Data (Figure S3). Our data of the over-expression of CIAPIN1 in the sera of CCA patients was in accordance with the data of bioinformatic analysis of CIAPIN1 expression in CCA tissue compared with normal samples obtained from the GEPIA2 database, as shown in Figure 1d.

### 3.3. The Correlation between Serum CIAPIN1 Levels and Clinical Parameters of CCA Patients

**Figure 5:**
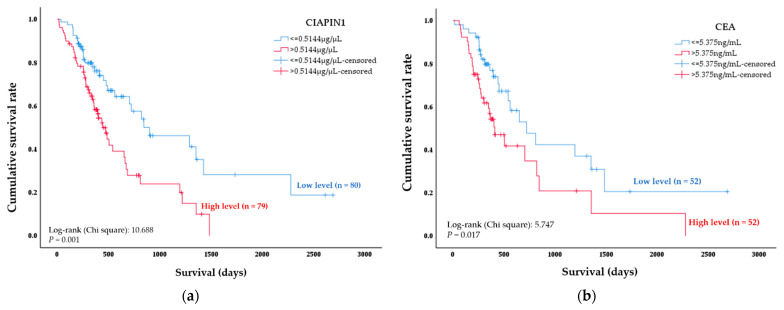

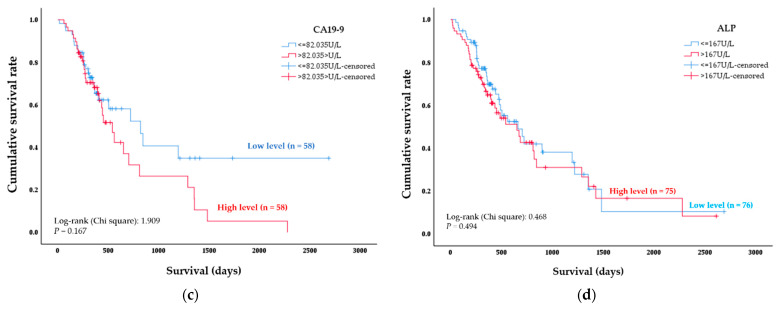
Kaplan–Meier plots showing the comparison of overall survival time of CCA patients according to the serum levels of (**a**) CIAPIN1, (**b**) CEA, (**c**) CA19-9, and (**d**) ALP. The curves show the overall survival of CCA patients having high (red line) and low (blue line) serum levels. A significant difference in the survival time was observed between high- and low-serum biomarker level groups by log-rank test and *p* < 0.05.

To examine the clinical importance of CIAPIN1, the relationship between CIAPIN1 and clinicopathological features of CCA patients were investigated. At first, tests of normality revealed that the data were non-normally distributed (*p* < 0.0001, Kolmogorov–Smirnov Test); hence, the median CIAPIN1 expression value of the CCA group was used as a cut-off. These 159 CCA patients were divided into low (n = 80) and high (n = 79) CIAPIN1 expression groups. Chi-square analysis revealed that high CIAPIN1 expression was significantly correlated with lymph node metastasis (*p* = 0.024) and overall survival (OS) (*p* = 0.037). However, there was no correlation between CIAPIN1 expression and patients’ age, gender, total bilirubin, direct bilirubin, ALT, AST and ALP (Table 2). Next, the log-rank test with Kaplan–Meier estimates was adopted to determine whether CIAPIN1 is a significant prognostic marker for the survival of patients with CCA. The results showed that the overall survival of CCA patients with high serum CIAPIN1 level was significantly shorter than that of the patients with the low serum CIAPIN1 level, with the median estimations of survival time of 441 ± 49 days (95% CI: 344–538) and 898 ± 289 days (95% CI: 332–1464), respectively; *p* = 0.001 (Figure 5a). This result is compatible with the bioinformatics analysis, as shown in Figure 2c. We next performed Cox’s univariate and multivariate hazard regression model to identify the independent factors that were significantly associated with OS to examine the effect of the covariates including the patients’ CIAPIN1 expression level, lymph node metastasis, age, sex, and other factors. The results showed that the serum CIAPIN1 level was recognized as an independent prognostic factor for the overall survival of CCA patients (HR = 1.88, 95% CI: 1.123–3.141; *p* = 0.016), which was the same as lymph node metastasis (Table 3).

### 3.4. Potential Prognostic Predictivity of Serum CIAPIN1 and Other Prognostic Markers

To compare the potential prognostic predictivity of serum CIAPIN1 and currently used markers, firstly, the Spearman analysis was performed to examine the correlation between CIAPIN1 and CEA, CA19-9 or ALP. The results indicated that the serum level of CIAPIN1 was not correlated with the serum level of CEA, CA19-9, or ALP (Figure S4). Then, the Kolmogorov–Smirnov test was carried out to verify the distributed data. Similar to the CIAPIN1 data, CEA, CA19-9 and ALP data were non-normally distributed; therefore, we used their median values as a cut-off for the CCA group. A log-rank test with a Kaplan–Meier estimate was performed to determine whether each of them can be a significant prognostic marker for the overall survival time of CCA patients (Figure 5b–d). The results showed that higher serum CIAPIN1 level (Chi-square: 10.688, *p* = 0.001) was associated with shorter survival, more significantly than those of CEA, CA19-9, and ALP (5.747 and 0.017; 1.909 and 0.167; 0.468 and 0.494, respectively).

## 4. Discussion

Nowadays, bioinformatics plays a key role in the biomarker discovery process, bridging the gap between initial discovery phases and experimental phases. In the present study, 281 mitochondrial proteins of CCA and adjacent tissues were re-analyzed using bioinformatic tools. CIAPIN1 was identified as a potential prognosis biomarker for CCA. CIAPIN 1, also known as anamorsin, is an anti-apoptotic molecule which has no sequence similarities to a series of apoptosis-associated molecules such as Bcl-2 or caspase family members. It is a key mediator of RAS signaling pathways and mediates the maintenance of hematopoiesis in the fetal liver [21]. CIAPIN1 was reported as a candidate indicator for diagnosis and prognosis for several human cancers and is proposed as a therapeutic target for anticancer interventions [22]. However, the biological function of CIAPIN1 in CCA has not been studied, nor has its expression in the sera of CCA patients been determined. Hence, the measurement of serum CIAPIN1 level in CCA patients has clinical importance.

In this study, we showed that CIAPIN1 is a secretory protein via an unconventional secretory pathway. In general, most secretory proteins, which have signal peptides, travel through a well-documented conventional secretion pathway involving the endoplasmic reticulum (ER) and the Golgi complex [23]. In the present study, by bioinformatic analysis, SignalP predicted that CIAPIN1 lacks a signal peptide, indicating that this protein is not secreted through a conventional secretion pathway. However, the SecretomeP results showed that CIAPIN1 protein can be a secretory protein via an unconventional secretory pathway. In this pathway, the proteins reach the plasma membrane or extracellular space via unconventional routes that do not bear a signal peptide or via a route that bypasses the Golgi [23]. This finding was confirmed by PPD because CIAPIN1 is listed in PPD. Obviously, CIAPIN1 has been expressed in various tissues, and several reports showed that the expression of CIAPIN1 protein in tumor tissue is associated with cancer prognosis [24,25,26,27]. However, its expression levels in the sera of patients with diseases, including CCA, is still lacking. Our results reported here are the first to have measured CIAPIN1 levels in the sera of CCA patients and healthy controls.

CIAPIN1 protein is encoded by the *CIAPIN1* gene located in the chromosome 16q21, produced in the cytoplasm, and is localized in both the cytoplasm and mitochondria (UniProt KB). In addition, Hao et al. (2006) demonstrated the localization of CIAPIN1 in the nucleus [28]. A previous study of our group demonstrated CIAPIN1 and another four proteins are uniquely detected in the mitochondrial fraction of CCA tissues [10]. Several studies have reported that mitochondrial proteins could be potential biomarkers for cancer. Heat shock proteins (HSPs) are a large family of chaperones, and mitochondrial chaperones have been used as serum markers for cancers. For instance, HSP60 is primarily localized in the eukaryotic mitochondria and its overexpression in the sera is used as a biomarker for the diagnosis and prognosis of several cancers, including colorectal and breast cancers [29,30]. Recently, two mitochondrial proteins—PDK3 and AIFM3—in the sera have been determined and can be used as a prognostic marker for CCA [11,12]. The present results showed that CIAPIN1 is also a mitochondrial protein marker in the sera of CCA patients. In this study, our data showed that serum CIAPIN1 levels in the CCA patients were significantly higher than those of HC (*p* < 0.0001). In addition, the search on the GEPIA2 online database confirmed the high expression levels of the *CIAPIN1* gene in CCA tissues. This result from the GEPIA2 database clearly supports the results of our serum CIAPIN level measurement. Although the exact source of serum CIAPIN1 proteins remains unknown, higher serum CIAPIN1 levels of CCA patients might be attributed, at least in part, to CCA cells in tissues because CIAPIN1 was significantly expressed in CCA tumor compared with normal tissues (Figure 1d). The paired specimens of serum and CCA tissues of the patients and CCA cell line as well should be investigated further to ensure the secretory protein nature of CIAPIN1.

In the present study, the GEPIA2 revealed that higher CIAPIN1 expression in CCA tissues was correlated with shorter disease-free survival time (Figure 2c). Furthermore, our dot-blot quantification data showed that a higher serum CIAPIN1 level was correlated with a shorter overall survival time of CCA patients (Figure 5a), indicating consistency with the bioinformatic analysis. In particular, the log-rank test with a Kaplan–Meier survival curve revealed that the overall survival of CCA patients with a high serum CIAPIN1 level was significantly shorter than those with a low serum CIAPIN1 level. In addition, using a Cox proportional hazards model for univariate and multivariate analyses, CIAPIN1 was identified as an independent prognostic biomarker for CCA patients. Moreover, in comparison of CIAPIN1 and current CCA biomarkers for their potential predictivity of survival time of CCA patients by log-rank analysis, CIAPIN1 was superior to CEA, CA19-9 and ALP for prognostic prediction of CCA patients. The concordant findings of high expression of CIAPIN1 in tumor tissues and association to shorter survival have also been reported in other cancers; for example, CIAPIN1 was an independent prognostic factor for epithelial ovarian cancer and ovarian serous carcinoma, in that the increase of CIAPIN1 expression has been significantly associated with the poor clinical outcome of cancer patients [24,31]. Likewise, the decrease of CIAPIN1 expression in colorectal cancer, pancreatic cancer, and non-small cell lung carcinoma has been significantly associated with the clinical outcome of cancer patients; patients with weak or negative CIAPIN1 expression are always associated with longer survival duration [25,26,27].

In terms of CIAPIN1 and tumor metastasis, there are some research papers mentioning on this issue. Cho et al. (2012) found that CIAPIN1 is required for RhoGD12-mediated invasion [32]. Related to this, Nymoen et al. (2015) revealed that CIAPIN1 mRNA expression was significantly related to shorter overall survival of patients with metastatic serous ovarian carcinoma [24]. Our data showed that the serum CIAPIN1 level was significantly higher in CCA patients with lymph node metastasis than in the patients without metastasis (Figure S5). These findings suggest that an increase of CIAPIN1 expression might play a potential role in promoting the progression of CCA. The function of CIAPIN1 in the invasion and migration of CCA requires further investigation in the future.

## 5. Conclusions

In conclusion, the present study identified CIAPIN1 as a potential prognosis biomarker from our mitochondrial proteome database for CCA patients by bioinformatics. In particular, the higher serum level of CIAPIN1 was associated notably with lymph node metastasis and shorter overall survival time. Moreover, CIAPIN1 was identified as a superior indicator to CEA, CA19-9 and ALP for the prognostic prediction of CCA patients. Therefore, the serum CIAPIN1 level can be used as a biomarker for the poor prognosis of CCA patients.

## Figures and Tables

**Table 1:** Characteristics of cholangiocarcinoma patients and healthy controls.

Parameters (Normal Range)	Cholangiocarcinoma	Healthy Controls	*p*-Value
	n = 159	n = 93	
Median age	61 ± 6 ^g^	41 ± 8 ^b^	<0.0001 *
Age range (Years)	(31–78)	(19–85)	
Total protein	7.4 ± 0.5 ^f^	NA	NA
(6.5–8.8 g/dL)	(4.6–10.0)		
Total bilirubin	0.60 ± 1.00 ^f^	NA	NA
(0.25–1.5 mg/dL)	(0.20–32.00)		
Direct bilirubin	0.3 ± 0.8 ^e^	NA	NA
(0–0.5 mg/dL)	(0–22.5)		
ALT	41 ± 20 ^f^	23 ± 8 ^a^	<0.0001 *
(4–36 U/L)	(1–795)	(4–172)	
AST	42 ± 18 ^f^	21 ± 5 ^a^	<0.0001 *
(12–32 U/L)	(4–1112)	(6–72)	
ALP	167 ± 82 ^f^	48 ± 9 ^a^	<0.0001 *
(42–121 U/L)	(35–1068)	(1–98)	
CA19–9	82 ± 436 ^d^	NA	NA
(0–37 U/mL)	(0.6–1000)		
CEA	5.3 ± 5.7 ^c^	NA	NA
(0–2.5 ng/mL)	(0.9–917.6)		

Value shows median ± quartile deviation (min–max); a, b, c, d, e, f, and g represent the number of analyzed samples = 91, 92, 104, 116, 150, 151, and 159, respectively; NA = not analyzed; ALT = alanine transaminase; AST = aspartate transaminase; ALP = alkaline phosphatase. * Significant difference between HC and CCA of each clinical parameter was calculated by Mann–Whitney U test.

**Table 2:** Correlations between clinicopathological features and serum CIAPIN1 levels of CCA patients.

Patient Characteristics	Serum CIAPIN1 Level (µg/µL)		*p*
	Low (≤0.5144)	High (>0.5144)	
Gender			^a^ 1.000
Male	55 (34.6%)	54 (34%)	
Female	25 (15.7%)	25 (15.7%)	
Age (median range)			^a^ 0.429
≤61 years	39 (24.5%)	44 (27.7%)	
>61 years	41 (25.8%)	35 (22%)	
Lymph node metastasis			^a^ 0.024 *
No	50 (32.0%)	34 (21.8%)	
Yes	29 (18.6%)	43 (27.6%)	
Survival time (median range)			^a^ 0.037 *
≤360 days	50 (31.4%)	62 (39%)	
>360 days	30 (18.9%)	17 (10.7%)	
Total protein (g/dL)	7.40 ± 0.42	7.50 ± 0.70	^b^ 0.379
	(n = 77)	(n = 74)	
Total bilirubin (mg/dL)	0.60 ± 0.80	0.65 ± 1.06	^b^ 0.173
	(n = 77)	(n = 74)	
Direct bilirubin (mg/dL)	0.20 ± 0.64	0.30 ± 1.04	^b^ 0.140
	(n = 76)	(n = 74)	
ALT (U/L)	37 ± 20.75	44 ± 21	^b^ 0.145
	(n = 77)	(n = 74)	
AST (U/L)	41 ± 14.25	46 ± 19.63	^b^ 0.094
	(n = 77)	(n = 74)	
ALP (U/L)	160 ± 82.50	185 ± 74.75	^b^ 0.108
	(n = 77)	(n = 74)	

* Statistically significant correlation as *p* < 0.05; ^a^ Fisher’s exact test; to these variables, the difference between high- and low-CIAPIN1 groups were estimated using ^b^ Mann–Whitney U test. Data represent median ± quartile deviation. Median of serum CIAPIN1 level in the CCA group was used to separate between low and high groups.

**Table 3:** Univariate and multivariate Cox regression analyses of clinicopathological parameters and serum CIAPIN1 levels of CCA patients.

Clinicopathological Factors	Univariate Analysis				Multivariate Analysis			
	n	HR	95% CI	*p*	n	HR	95% CI	*p*
Gender				0.388				0.326
Male	109	1.00	-		99	1.00	-	
Female	50	1.34	0.838–2.133		48	1.29	0.776–2.148	
Age (year, median range)				0.869				0.430
≤61	83	1.00	-		78	1.00	-	
>61	76	0.96	0.616–1.507		69	1.22	0.743–2.007	
Lymph node metastasis				<0.0001 *				<0.0001 *
No	84	1.00	-		80	1.00	-	
Yes	72	2.91	1.819–4.695		67	2.75	1.653–4.562	
Total protein (g/dL)				0.618				0.996
Normal (6.5–8.8 g/dL)	123	1.00	-		121	1.00	-	
Abnormal (<6.5 or >8.8 g/dL)	28	1.15	0.672–1.951		26	1.00	0.555–1.796	
Total bilirubin (mg/dL)				0.666				0.894
Normal (0.5–1.5 mg/dL)	51	1.00	-		50	1.00	-	
Abnormal (<0.5 or >1.5 mg/dL)	100	0.90	0.558–1.451		97	0.96	0.557–1.668	
Direct bilirubin (mg/dL)				0.318				0.060
Normal (≤0.5 mg/dL)	93	1.00	-		92	1.00	-	
Abnormal (>0.5 mg/dL)	57	0.78	0.481–1.268		55	0.57	0.315–1.023	
ALT (U/L)				0.622				0.825
Normal (4–36 U/L)	65	1.00	-		63	1.00	-	
Abnormal (>36 U/L)	86	1.12	0.707–1.785		84	1.09	0.521–2.466	
AST (U/L)				0.378				0.652
Normal (12–32 U/L)	54	1.00	-		52	1.00	-	
Abnormal (>32 U/L)	97	1.24	0.766–2.020		95	1.18	0.568–2.466	
ALP (U/L)				0.925				0.760
Normal (42–121 U/L)	49	1.00	-		48	1.00	-	
Abnormal (>121 U/L)	102	1.02	0.627–1.673		99	0.92	0.536–1.577	
CIAPIN1				0.001 *				0.016 *
Low (≤0.5144 µg/µL)	80	1.00	-		75	1.00	-	
High (>0.5144 µg/µL)	79	2.13	1.34–3.38		72	1.88	1.123–3.141	

Abbreviations: HR = hazard ratio; CI = confidence interval. * Statistically significant as *p* < 0.05.

## Supplementary Materials

The following are available online at https://www.mdpi.com/article/10.3390/diagnostics11061054/s1. Figure S1: Detection of CIAPIN1 protein in HC and CCA sera by the Western blot using rabbit polyclonal antibody against human CIAPIN1 (Cat#orb377996, Biorbyt, Cambridge, UK); Figure S2: Preparation of CIAPIN1 standard curve for dot blot assay; Figure S3: The representative dot blot images of CIAPIN1 levels from Amersham imager 600-analyzer; Figure S4: The Spearman’s correlations tested between the levels of serum CIAPIN1 and (a) CEA, (b) CA19-9, or (c) ALP; Figure S5: The plot chart shows the serum CIAPIN1 levels of metastatic and non-metastatic CCA patients.
