# Supplementary material for: Serum Levels of Cytokine-Induced Apoptosis Inhibitor 1 (CIAPIN1) as a Potential Prognostic Biomarker of Cholangiocarcinoma
Source: Diagnostics (Basel). 2021 Jun 8;11(6):1054. doi: 10.3390/diagnostics11061054 (PMC8227425; doi:10.3390/diagnostics11061054)
Supplement: Supplementary file 1 [file diagnostics-11-01054-s001.zip › diagnostics-1232456-s001.pdf]

diagnostics

MDPI

Article

Serum Levels of Cytokine-Induced Apoptosis Inhibitor 1 (CIAPIN1) as a Potential Prognostic Biomarker of Cholangiocarcinoma

Son Dinh An Truong [1], Doungdean Tummanatsakun [1], Tanakorn Proungvitaya [1], Temduang Limpaiboon [1,2], Molin Wongwattanakul [1,3], Daraporn Chua-on [1], Sittiruk Roytrakul [4] and Siriporn Proungvitaya [1,3,*]

[1] Centre of Research and Development of Medical Diagnostic Laboratories, Faculty of Associated Medical Sciences, Khon Kaen University, Khon Kaen 40002, Thailand; sontda@kkumail.com (S.D.A.T.); pui_ddlab41@hotmail.com (D.T.); tanakorn@kku.ac.th (T.P.); temduang@kku.ac.th (T.L.); moliwo@kku.ac.th (M.W.); chuaondaraporn@kkumail.com (D.C.-o.)
[2] Cholangiocarcinoma Research Institute, Faculty of Medicine, Khon Kaen University, Khon Kaen 40002, Thailand
[3] Center for Innovation and Standard for Medical Technology and Physical Therapy, Faculty of Associated Medical Sciences, Khon Kaen University, Khon Kaen 40002, Thailand
[4] National Center for Genetic Engineering and Biotechnology, National Science and Technology Development Agency, Pathumthani 12120, Thailand; sittiruk@biotec.or.th
* Correspondence: sirpat@kku.ac.th; Tel.: +66-4-3202088

Citation: Truong, S.D.A.; Tummanatsakun, D.; Proungvitaya, T.; Limpaiboon, T.; Wongwattanakul, M.; Chua-on, D.; Roytrakul, S.; Proungvitaya, S. Serum Levels of Cytokine-Induced Apoptosis Inhibitor 1 (CIAPIN1) as a Potential Prognostic Biomarker of Cholangiocarcinoma. *Diagnostics* **2021**, *11*, 1054. https://doi.org/10.3390/diagnostics11061054

Received: 7 May 2021
Accepted: 3 June 2021
Published: 8 June 2021

Publisher's Note: MDPI stays neutral with regard to jurisdictional claims in published maps and institutional affiliations.

Copyright: © 2021 by the authors. Licensee MDPI, Basel, Switzerland. This article is an open access article distributed under the terms and conditions of the Creative Commons Attribution (CC BY) license (https://creativecommons.org/licenses/by/4.0/).

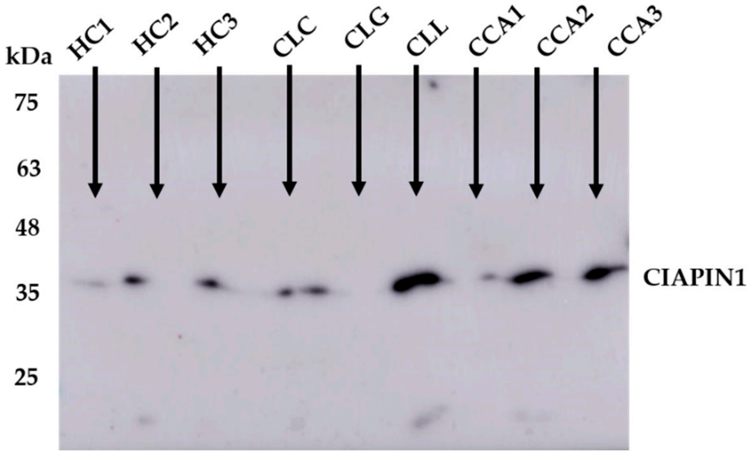

Figure S1. Detection of CIAPIN1 protein in HC and CCA sera by the Western blot using rabbit polyclonal antibody against human CIAPIN1 (Cat#orb377996, Biorbyt, Cambridge, UK). HC: Healthy control; CLC: Chronic cholecystitis; CLG: Chronic cholangitis; CLL: Cholelithiasis; CCA: Cholangiocarcinoma.

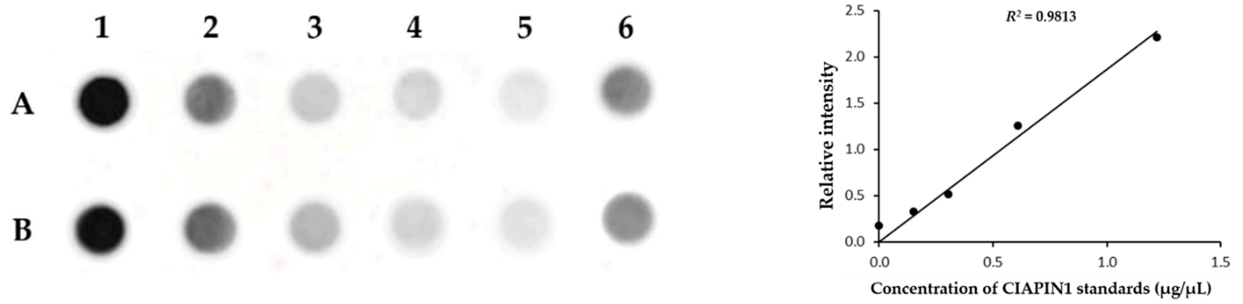

(**a**) (b)

Figure S2. Preparation of CIAPIN1 standard curve for dot blot assay. (**a**) Samples A1 to A4 were serially diluted standards, sample A5 the blank control and sample A6 pooled sera of CCA patients. Row B was the duplicate of row A. (**b**) The standard curve of CIAPIN1 levels.

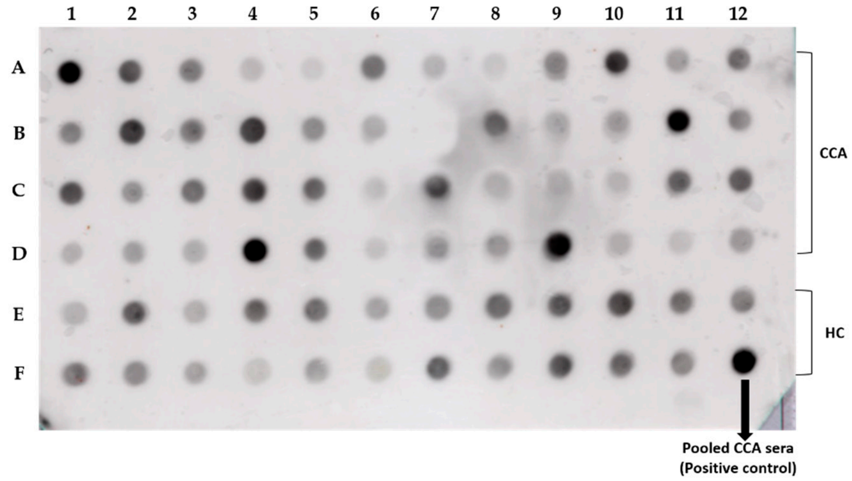

Figure S3. The representative dot blot images of CIAPIN1 levels from Amersham imager 600 analyzer. The spots were presented in order along the horizontal lines. A spot at the right lower corner was a positive control (pooled CCA sera). The first four lines were CCA sera, and the last two lines were HC serum samples. HC was undiluted (neat); The CCA sera was diluted 1:2 by normal saline for an investigation of serum CIAPIN1 expression.

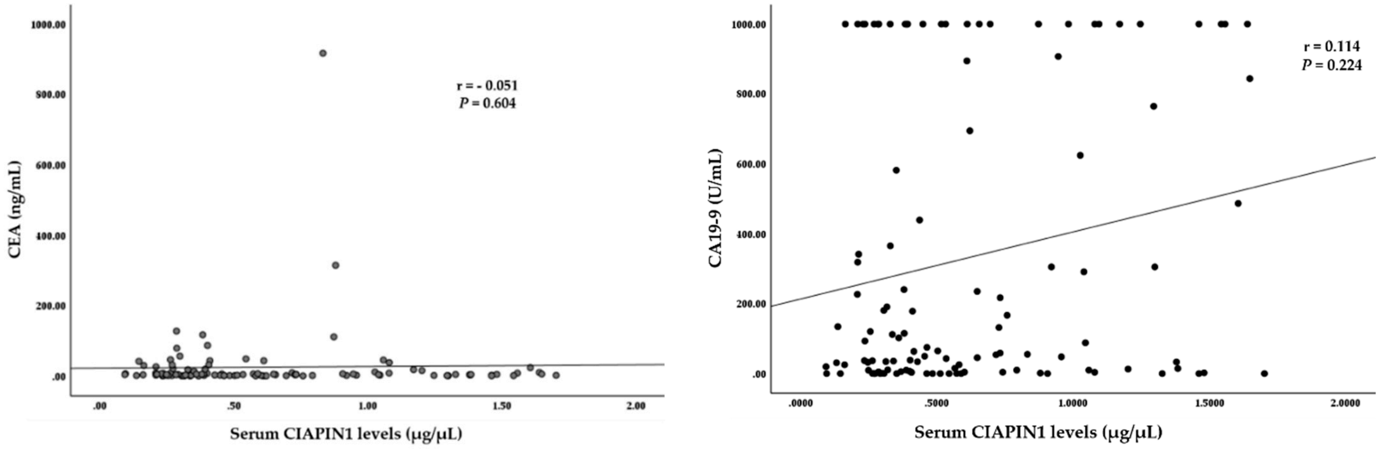

(**a**) (**b**)

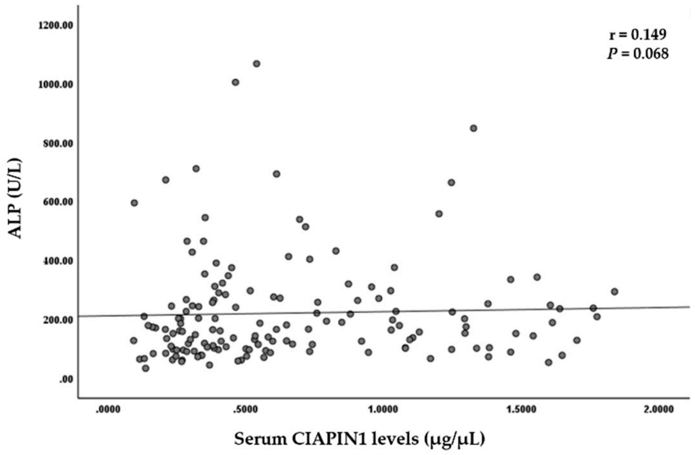

(**c**)

Figure S4. The Spearman's correlations tested between the levels of serum CIAPIN1 and (**a**) CEA; (**b**) CA19-9; or (**c**) ALP. The results showed no correlations between the serum CIAPIN1 level and either CEA, CA19-9, or ALP. $p < 0.05$ as significance.

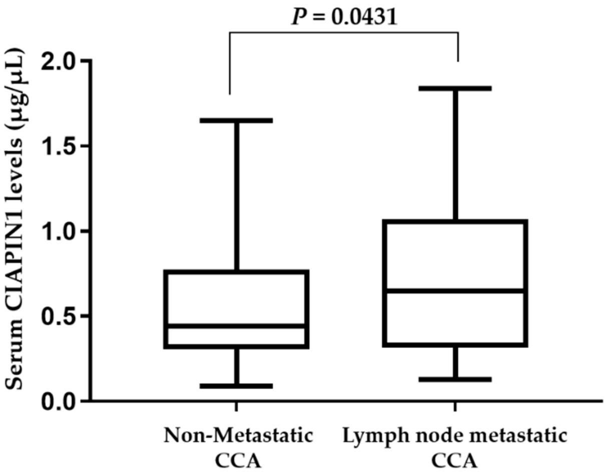

Figure S5. The plot chart shows the serum CIAPIN1 levels of lymph node metastatic and non-metastatic CCA patients, a significant difference as $p < 0$ .05 by Mann-Whitney U test.
